# Global mapping of randomized controlled trials in dentistry

**DOI:** 10.1590/0103-644020246233

**Published:** 2024-12-16

**Authors:** Mayara Colpo Prado, Lara Dotto, Bernardo Agostini, Rafael Sarkis-Onofre

**Affiliations:** 1 Graduate Program in Dentistry, ATITUS Educação, Passo Fundo, RS, Brazil.; 2 School of Dentistry, Regional Integrated University of High Uruguay and Missions, Erechim, RS, Brazil.

**Keywords:** dentistry, global mapping, meta-research, randomized controlled trials, scientific collaboration

## Abstract

This meta-research sought to evaluate the conduct, reporting, and main characteristics of published randomized controlled trials (RCTs) in dentistry. A search was performed on PubMed for RCTs in dentistry indexed from 31 December 2016 to 31 December 2021. Two reviewers independently screened the studies for the presence of eligibility criteria. Only studies in English were considered. Journal and author data, subject, citation and publishing metrics, reporting, and details of study conduct were collected. A descriptive analysis of the data, a map depicting the number of RCTs per country, and a network graph of scientific collaboration among different countries were presented. We included 844 articles. The main research area was periodontics (16.35%). The highest number of RCTs was attributed to Brazil (16.59%). Authors from the USA established the most links with other countries. Most studies did not report the use of CONSORT (67.89%), the type of randomization (69.31%), or the mechanism used to implement the random allocation sequence (52.37%). However, most studies included “randomized” in the title (71.92%) and reported the method for generating a random allocation sequence (65.88%) and blinding (74.88%). RCTs are the basis for clinical decision-making. Our results provide a better view of current RCTs and identify areas that require improvement. Brazil was the country that produced the most RCTs, and the USA was the main collaborator. We emphasize the variability of reporting characteristics and study conduct.

## Resumo

Esta meta-pesquisa buscou avaliar a conduta, o relato e as principais características de ensaios clínicos randomizados (ECRs) publicados na odontologia. Foi realizada uma busca no PubMed por ECRs em odontologia indexados de 31 de dezembro de 2016 a 31 de dezembro de 2021. Dois revisores avaliaram independentemente os estudos quanto à presença dos critérios de elegibilidade. Apenas estudos em inglês foram considerados. Dados do periódico e do autor, especialidade, métricas de citação e publicação, detalhes de relato e conduta dos estudos foram coletados. Uma análise descritiva dos dados, um mapa mostrando o número de ECRs por país e um gráfico de rede da colaboração científica entre diferentes países foram apresentados. Incluímos 844 artigos. A principal área de pesquisa foi a periodontia (16,35%). O maior número de ECRs foi atribuído ao Brasil (16,59%). Autores dos EUA estabeleceram a maioria das conexões com outros países. A maioria dos estudos não relatou o uso do CONSORT (67,89%), o tipo de randomização (69,31%) ou o mecanismo usado para implementar a sequência de alocação randomizada (52,37%). No entanto, a maioria dos estudos incluiu "randomized" no título (71,92%) e relatou o método para gerar uma sequência de alocação randomizada (65,88%) e o cegamento (74,88%). Os ECRs são a base para a tomada de decisões clínicas. Nossos resultados fornecem uma visão mais clara dos ECRs atuais e identificam áreas que requerem melhorias. O Brasil foi o país que mais produziu ECRs, e os EUA foram o principal colaborador. Ressaltamos a variabilidade das características de relato e conduta do estudo.

## Introduction

The American Dental Association defines evidence-based dentistry as “an approach to oral health care that requires the judicious integration of systematic assessments of clinically relevant scientific evidence, relating to the patient’s oral and medical condition and history, with the dentist’s clinical expertise and the patient’s treatment needs and preferences” ^1^. Recently, this concept has become more popular and has gained importance. The need for predictable and effective dental treatments has led clinicians to seek validated therapeutic approaches to support their clinical decisions. One of the most powerful types of evidence used in decision-making originates from randomized controlled trials (RCTs) since they are considered the “gold standard” for evaluating health interventions ^2^ ^,^ ^3^.

Every year, an impressive number of systematic reviews and RCTs are published, which suggests that the value of these methods is being recognized ^4^ ^,^ ^5^. A recent study that evaluated trends in clinical research literature from 1991 to 2020 found that the annual RCT growth rate maintained a steady upward trend until 2017 but with slight fluctuation over the last three years of evaluation. In 1991, 2037 RCTs were published, while in 2020, there were 17,415, highlighting a substantial increase in this type of study over time ^5^. Despite the recent increase in published RCTs, the number of RCTs in dentistry is still considerably lower than in the medical field, demonstrating a deficit in evidence-based research in dentistry. In 2017, only 533 RCTs in dentistry were indexed in PubMed ^6^.

The publication of an RCT does not guarantee its quality. Additionally, studies suggest that articles with a high level of evidence are not consistently associated with the impact factor of the scientific journal in which they are published ^7^ ^,^ ^8^. High-quality RCTs with reliable results and an impact on clinical practice must be well planned, conducted, and reported to avoid serious harm to patients, dentists, and the academic and scientific community. Thus, in recent years, initiatives have been developed to improve the quality of these studies, such as encouraging the protocol registration of RCTs, reporting guidelines such as CONSORT (Consolidated Standards of Reporting Trials Statement), and tools for assessing the risk of bias such as RoB 2 ^9^ ^,^ ^10^ ^,^ ^11^.

Among these initiatives, CONSORT is the most endorsed, widely cited, and recognized as one of the main milestones of the last century in health research methods ^12^ ^,^ ^13^. CONSORT has been available since 1996 to guide researchers in reporting RCTs systematically through a checklist of essential items that should be included in RCT reports to make them as complete and transparent as possible ^14^. In addition, the checklist serves as a method of evaluating the report and interpreting the study critically. Today, CONSORT has several extensions, and it was last updated in 2010 ^9^ ^,^ ^13^. Over time, evidence of the impact of CONSORT has accumulated, and studies have already shown that its endorsement by journals improves the quality of RCT reports in dentistry ^6^; however, several studies in different dental specialties indicate a need for improvement ^8^ ^,^ ^15^ ^,^ ^16^ ^,^ ^17^ ^,^ ^18^.

Our initial published analysis identified a gender gap in RCTs in dentistry, which is present in study authorship and collaboration between authors ^19^. However, considering the relevance of RCTs for evidence-based dentistry, it is necessary to understand the current characteristics of these studies, and how they were conducted and reported. Identifying improvements and gaps in this type of study is essential for the advancement of quality scientific knowledge and for the process of "transforming" evidence into clinical practice. Thus, this study aimed to evaluate the conduct, reporting, and main characteristics of recently published RCTs in dentistry.

## Materials and Methods

The meta-research study protocol was registered on the Open Science Framework and is available at the following link: https://osf.io/qbg9n/

### Eligibility criteria

As an inclusion criterion, the study needed to be an RCT as described by Friedman et al. ^2^. Furthermore, the RCTs should be in the dental field, that is, related to the evaluation, diagnosis, prevention, and/or treatment of diseases, disorders, and/or conditions of the oral, maxillofacial, and/or adjacent area and associated structures or that discussed educational aspects. Articles indexed from 31 December 2016 to 31 December 2021 were included, regardless of the topic (e.g., epidemiological, therapeutic, or diagnostic), methods, or level of detail reported. However, as an exclusion criterion, articles published in languages other than English were excluded due to a lack of funding for article translation.

### Search

Box 1presents the search strategy used. We performed searches in PubMed, based on MeSH terms, for RCTs indexed from 31 December 2016 to 31 December 2021.

**Box 1:**
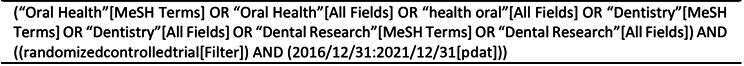
Search Strategy for PubMed

### Screening

Search results were transferred to DistillerSR (DistillerSR, Evidence Partners Incorporated, ON, CA), an online software that automates screening and data extraction. A pilot test with 20 randomly selected studies was performed using the screening form. First, two researchers independently evaluated titles and abstracts for the presence of eligibility criteria. Articles were classified as “include,” “exclude,” or “uncertain.” Second, an additional eligibility screening was performed using the full text of the records classified as “include” and “uncertain.” This screening was performed independently by the same two reviewers. Discrepancies in evaluating titles, abstracts, and full texts were resolved by consensus or, in the absence of consensus, the opinion of a third reviewer.

### Sample

The sample in this study is part of a larger project, and it is the same as that used in our previous study, which evaluated women's participation in science ^19^. We anticipated that around 2,500 RCTs would be identified based on a previous study ^6^; thus, the minimum sample size to find associations, considering an error probability of 5% (α = 0.05), power (1-β) of 80%, an equal proportion of exposed and unexposed (women and men), and an estimated effect size of odds ratio (OR) = 1.5 based on a previous study of female team contribution ^20^, was 844 studies. We used an Excel list of random numbers containing all articles classified as included to randomly selected 844 studies considering the proportion of articles indexed per year. Only the most recent report was used if multiple reports from the same study were identified.

### Data extraction

A standardized data extraction form was created in DistillerSR. A pilot test was conducted through discussion among the three reviewers to ensure consistency in interpreting the items. Twenty of the 844 included studies were selected for the pilot test using a list of random numbers in Microsoft Excel. Two reviewers extracted half of all included studies, and another reviewer verified the data extraction and consistency of interpretation. In cases of doubt or inconsistency, the data were re-extracted.

All collected data are in our protocol available on the Open Science Framework. However, for this study, we extracted the following data: journal, impact factor (year 2022), type of journal access, subject of article (based on dental specialties recognized by the Federal Council of Dentistry of Brazil ^21^), number of authors, country of corresponding author, country of the first ten authors of each article, total number of citations, and weighted relative citation ratio (wRCR) as reported by the iCite tool (https://icite.od.nih.gov) of each article included. The wRCR was considered a measure of influence, with higher values representing the most cited publications ^22^.

In addition, we collected the following information on study reporting and conduct from the main points of the CONSORT statement: whether the use of the CONSORT guideline was reported and whether it was reported appropriately (i.e., as a tool to guide study reporting, not to assess the methodological quality of studies or determine how to design and conduct studies); the presence or absence of the term “randomized” in the title; the type of study design and whether it was reported; how many centers were involved and whether this information was reported; number of study groups; and the type of randomization, methods used to generate and implement the allocation sequence, and type of blinding and whether this information was reported. In other words, the reporting of RCTs was assessed based on whether or not the authors mentioned the aspects assessed in our studies. The conduct in RCTs was assessed based on what the authors reported about the methodological aspects of the study.

### Data analysis

All descriptive analyses were performed in Microsoft Excel using frequency for categorical data and mean and standard deviation for continuous data. Using Microsoft Excel, we prepared a map depicting the number of RCTs by country of the corresponding authors. The darker the color of a country in the chart, the more RCTs were assigned to that country.

Network graphs were generated in the bibliometric software VOSviewer (version 1.6.19) from an Excel spreadsheet detailing the scientific collaboration among countries based on the corresponding author of each of the articles. Only the first ten authors of each article were included in the analysis. We defined a cross-country collaboration as an article for which the country of the corresponding author differed from that of any of the other authors. The sizes of the circles are proportional to the total link strength between a given country and other countries. The colors of the circles represent the continents to which the countries belong (America: blue, Asia: yellow, Africa: purple, Europe: green, Oceania: red). The lines represent links between countries, and their thickness represents the strength of the connection.

## Results

Through the PubMed search, we identified 5,557 studies, and 3,512 met the eligibility criteria. Of these, 844 studies were included in analyses, as suggested by the sample size calculation. More details of these steps can be found in our previous study ^19^.

Table 1 presents the main characteristics of the included studies. The 844 articles were published in 195 journals. The journal with the most published articles included in our study was *Clinical Oral Investigations* (63, 7.46%), followed by two journals in implant dentistry, *Clinical Oral Implants Research* and *Clinical Implant Dentistry and Related Research* (34, 4.03%, and 30, 3.55%, respectively). Most journals had hybrid-type access (531, 62.91%), and a minority had subscription access (85, 10.07%). The impact factors of the journals in our study ranged from 0.863 to 24.897, with a mean of 2.980 (± 0.856). The main specialties were periodontology (138 articles, 16.35%) and oral and maxillofacial surgery (135, 16.00%). The average number of citations per article was 7.20 (± 7.40), and the average wRCR was 1.91 (± 1.75). The number of authors ranged from 1-47, with an average of 6.5 (± 2.12).

**Table 1:** Characteristics of the included studies

Journal*	N (%)
Clinical Oral Investigations	63 (7.46)
Clinical Oral Implants Research	34 (4.03)
Clinical Implant Dentistry and Related Research	30 (3.55)
BMC Oral Health	28 (3.32)
Journal of Clinical Periodontology	25 (2.96)
Journal of Dentistry	23 (2.73)
Journal of Oral and Maxillofacial Surgery	23 (2.73)
Photodiagnosis and Photodynamic Therapy	20 (2.37)
European Journal of Orthodontics	17 (2.01)
American Journal of Orthodontics and Dentofacial Orthopedics	15 (1.78)
Journal Impact (2022)	
Mean (±SD)	2.980 (±0.856)
Journal access	N (%)
Open access	223 (26.42)
Hybrid journal	531 (62.91)
Subscription journal	85 (10.07)
Subject (Federal Council of Dentistry of Brazil)	N (%)
Periodontics	138 (16.35)
Oral and Maxillofacial Surgery	135 (16.00)
Implantology	98 (11.61)
Restorative dentistry	95 (11.26)
Orthodontics	72 (8.53)
Pediatric Dentistry	58 (6.87)
Endodontics	55 (6.52)
Oral and Maxillofacial Pathology	29 (3.44)
Dental prosthesis	28 (3.32)
Public Health Dentistry	26 (3.08)
Temporomandibular Disorders and Orofacial Pain	21 (2.49)
Dentistry for Special Patients	16 (1.90)
Geriatric Dentistry	11 (1.30)
Jaw Facial Orthopedics	8 (0.94)
Other	51 (6.04)
Unclear	3 (0.35)
Article citations	
Mean (±SD)	7.20 (±7.40)
Weighted Relative Citation Ratio (wRCR)	
Mean (±SD)	1.91 (±1.75)
Number of authors	
Mean (±SD)	6.5 (±2.12)

*Top ten journals

Table 2 presents data on the reporting and conduct of the included RCTs. Most studies did not report the use of CONSORT (573, 67.89%), and of those that did, the majority reported its use inadequately (155, 18.37%). The term “randomized” was in the title of 71.92% (607) of included studies. However, most studies did not describe the trial design (399, 47.27%). Of those that did, the most frequent design was parallel (263, 31.16%). Additionally, 75.83% (640) of trials involved two study groups performed at a single center (542, 64.22%).

Most studies did not report the type of randomization (585, 69.31%). When it was reported, block randomization was the most frequent (144, 17.06%). A computer program or website was the most frequently used method for generating a random allocation sequence (394, 46.68%). Many studies reported using opaque, sealed envelopes or containers (296, 35.07%) to implement the randomized allocation sequence, but most did not report the mechanism used (442, 52.37%). Of blinding techniques, single blinding was the most used in the included RCTs (327, 38.74%).

**Figure 1:**
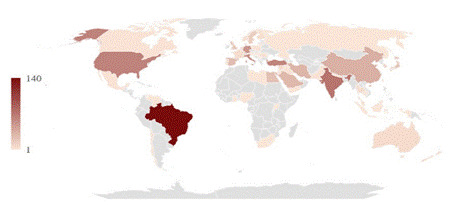
Map considering the number of RCTs by country of the corresponding authors. Dark colors represent countries with a higher number of RCTs.

Figure 1 depicts a map of the number of RCTs by country of the corresponding author. We identified 59 countries. The most significant number of trials was attributed to Brazil (140, 16.59%), followed by India (72, 8.53%), the USA (63, 7.46%), and Turkey (62, 7.35%).

**Figure 2:**
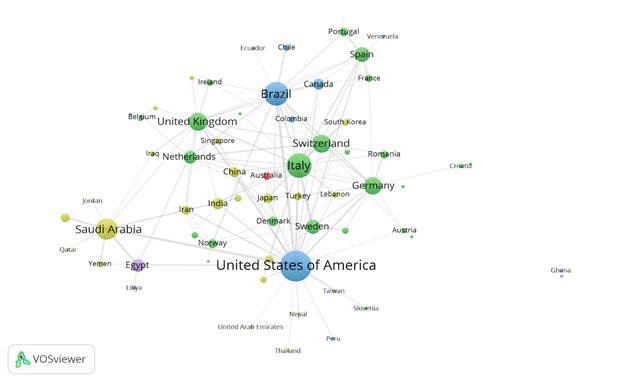
Collaboration network between countries, according to the authors' country, for all RCTs included in our study. A total of 62 connected countries (represented by circles) were included. The sizes of the circles are proportional to the total link strength between a given country and other countries. The lines represent links between countries, and their thickness represents the strength of the connection. The colors of the circles represent the continents: America: blue, Asia: yellow, Africa: purple, Europe: green, Oceania: red.

Figure 2 shows the cross-country collaboration network, according to the authors’ countries, for all RCTs included in our study. There were 62 countries represented by circles and 472 established connections. Authors from the USA established the most links with other countries (138, 29.24%), followed by Italy (87, 18.43%), Brazil (85, 18.01%), and Saudi Arabia (67, 14.19%). Similarly, authors from the USA collaborated the most with other countries ^26^, followed by Italy ^20^, the United Kingdom ^17^, and Brazil ^16^. The most frequent connections were between the USA and Brazil (28, 5.93%) and the USA and Saudi Arabia (24, 5.08%).

**Table 2:** Reporting and conduct characteristics

Reporting use of CONSORT	N (%)
Not reported the use	573 (67.89)
Appropriate use of the CONSORT	86 (10.19)
Inappropriate use of the CONSORT	155 (18.37)
Unclear	30 (3.55)
Reporting of “Randomized” in the title	N (%)
Not reported	237 (28.08)
Reported	607 (71.92)
Trial design	N (%)
Not reported	399 (47.27)
Parallel	263 (31.16)
Split mouth	120 (14.22)
Crossover	47 (5.57)
Factorial	7 (0.83)
Unclear	8 (0.95)
Study centers	N (%)
Not reported	209 (24.76)
Single	542 (64.22)
Multiple	89 (10.55)
Unclear	4 (0.47)
Number of study groups	N (%)
Two	640 (75.83)
Three	144 (17.06)
Four	44 (5.21)
More than four	16 (1.90)
The method used to generate the random allocation sequence	N (%)
Not reported	288 (34.12)
Computer software program/site	394 (46.68)
Coin	54 (6.40)
Random-numbers table	45 (5.33)
Drawing lots	11 (1.30)
More than one method	8 (0.95)
Unclear	9 (1.07)
Other	35 (4.15)
Type of randomization	N (%)
Not reported	585 (69.31)
Blocked randomization	144 (17.06)
Simple randomization	50 (5.92)
Stratified randomization	35 (4.15)
Blocked and Stratified randomization	24 (2.84)
Unclear	5 (0.59)
Other	1 (0.12)
The mechanism used to implement the random allocation sequence	N (%)
Not reported	442 (52.37)
Opaque, sealed envelopes/containers	296 (35.07)
Envelopes/containers (no further definitions)	36 (4.27)
Central randomization	14 (1.66)
Sequentially numbered	5 (0.59)
Unclear	14 (1.66)
Other	37 (4.38)
Blinding	N (%)
Not reported	212 (25.12)
Blind	327 (38.74)
Double-blind	216 (25.59)
Triple blind	34 (4.03)
Blinding not possible	49 (5.81)
Unclear	6 (0.71)

## Discussion

This is the first study to map recent dental RCTs globally. Our results highlight Brazil as the main source of RCTs in dentistry and the USA as a primary collaborator with other countries. In addition, we found that the reporting and conduct of RCTs are variable. Some practices, such as including “randomized” in the title, type of blinding, and method for generating the allocation sequence, seem to be commonly implemented. However, other important information, such as the use of CONSORT, type of randomization, and mechanism for implementing the allocation sequence, is often not appropriately reported, potentially jeopardizing the understanding of the article. Our study is important because it contributes to understanding the main characteristics of RCTs while highlighting those that deserve further exploration and development to improve this type of study. Moreover, publicly documenting shortcomings in reporting, conduct, and inequities in RCT production and collaboration across countries provides a means of assessing progress over the years.

Unsurprisingly, few RCTs and researchers were from countries with low and lower-middle incomes. Studies have shown that the productivity of biomedical research worldwide largely depends on each country’s gross national product per capita and expenditures allocated to research and development ^23^ ^,^ ^24^. However, we highlight the role of Brazil, a country with a medium-high income but the highest number of published dental RCTs. Other studies have also highlighted Brazil as an important source of scientific production in dentistry, especially for systematic reviews ^25^. Moreover, recently published data from 2022 in the Scimago Journal & Country Rank shows Brazil was the country that published the most international scientific articles in dentistry ^26^. Brazil is one of the countries with the fastest growth in academic dental production due to the large number of postgraduate programs in dentistry, in which evaluation processes, for a long time, encouraged maximization of article publication ^26^ ^,^ ^27^.

Although most included studies were conducted at a single center, which may result in a more significant treatment effect and risk of bias ^28^, we identified many cross-country collaborations. In 844 articles, 472 connections were established between authors from different countries. The map of collaborations is predominantly green, indicating a substantial presence of European countries. The USA played a central role in the collaborations identified in this study, corroborating a previous study in the biomedical field ^29^. Historically, medical research has had the most support in the USA, where, for many decades, more than half of the world’s funding has been generated. This advantage attracted many researchers to a better academic and scientific environment for many years. Also, many research projects in the USA are funded by private initiatives, unlike most other countries ^29^ ^,^ ^30^ ^,^ ^31^.

Many studies have attempted to evaluate the reporting of RCTs. A recent study analyzed 20,571 RCTs from biomedical research and found improvements in reporting since 1990. However, in 2015, 30.2% of RCTs were still poorly reported ^32^. In dentistry, many authors agree that reporting of RCTs is still suboptimal ^15^ ^,^ ^17^ ^,^ ^18^ ^,^ ^30^. Our findings corroborate this, particularly regarding details about randomization. The mechanism for implementing the allocation sequence was not reported in approximately 52% of the studies in our sample. Similar percentages of non-reporting were also observed in areas such as endodontics (40%)^15^, pediatric dentistry (64%)^8^, and restorative dentistry (60% to 83%)^16^ ^,^ ^17^.

The benefits of using CONSORT and the improvement that its endorsement and implementation provide to the reporting of RCTs have already been documented in the literature ^6^. In a recent study, 85% of studies did not report using CONSORT ^18^. Unfortunately, in our study, most also RCTs did not report using CONSORT, which could explain the insufficient reporting of many important items. The word limit stipulated by some journals may also restrict authors from detailing all the methodological characteristics used in a study and impair proper reporting based on CONSORT recommendations. In addition, many authors report certain information only at the request of journal reviewers or model their reporting on similar studies, even if they lack complete knowledge of a given method or type of study.

Our result indicated that most studies (71.92%) used the term “randomized” in the title. Similar results were reported in the evaluation of dental RCTs (64.2%) and RCTs on deep caries management (49.6%) ^6^ ^,^ ^18^. However, the term's presence in the title is insufficient to indicate that the study was designed, developed, and reported as an adequate RCT. Previous research found that only 39.6% of articles titled RCTs in high-impact dental journals were, in fact, RCTs ^33^. These data reaffirm that reporting an item does not always constitute adequate conduct. Most studies we analyzed that reported using CONSORT misreported its use, often by stating it was a guide for conduct rather than for reporting.

Methodological quality depends primarily on the degree to which a study’s design, conduct, and analysis meet the highest possible standards and reduce multiple potential biases ^34^. Nearly 60% of RCTs in biomedical research used inappropriate methods, according to a recent study ^32^. In dentistry, studies have shown that 40% to 52% of the RCTs included in their analyses presented a high risk of bias, indicating inappropriate conduct ^8^ ^,^ ^15^ ^,^ ^18^. In our sample, in addition to the reported inappropriate conduct regarding CONSORT, simple randomization was reported in approximately 6% of studies. However, the unpredictability of simple randomization can be disadvantageous. Simple randomization can produce highly disparate treatment arms in small sample sizes. In addition, simple randomization does not guarantee the control of important variables to be considered for both treatment groups, as occurs in stratified randomization ^3^ ^,^ ^35^. Furthermore, randomization through the flip of a coin was also observed in our sample. Because of the lack of randomness, difficulties of implementation in larger samples, and absence of an audit, it is recommended that researchers avoid using coin flips as a randomization mechanism ^35^.

Limitations of this study include the use of only one database, the non-evaluation of gray literature, and the inclusion of only articles in English, which may limit the generalization of the results. However, the database used includes the main journals in the field, most of which are published only in English. Data collection was not performed in duplicate, but we ensured data consistency by conducting a pilot test and involving a third reviewer in checking the collected data. The evaluation of the study reports was based on the main items present in the CONSORT statement; however, some items were not verified, and consequently, the completeness of the CONSORT was also not verified. Still, the evaluation of some RCT conduct characteristics was based only on the authors’ reports, and it was impossible to distinguish studies conducted with greater methodological rigor that were not reported or those studies that reported certain conduct but did not carry it out. Finally, the articles analyzed in our study were published until December 31, 2021, and the change in scenario to the present day must be considered. The authors believe that the data presented still reflect the current scenario and reinforce that it is commonly observed, in meta-research studies, a longer time between the methodological process of project execution and the publication of the article ^19^ ^,^ ^32^.

Despite the progress observed, considerable improvements in dental RCTs are necessary, and possible, and should be prioritized in future studies. These improvements are essential to ensure the quality of RCTs, avoid resource waste, and ensure that studies have a positive impact on global dental public health. Based on our findings, we suggest some future directions: 1) implement continuous training in courses and postgraduate programs to equip researchers with the best methodological practices to conduct good RCTs; 2) require authors to follow the CONSORT guidelines, including submitting the complete checklist with the manuscripts. The requirement can come from funders, journals, and reviewers and has the role of increasing knowledge and use of the guide, completeness of the report, and increasing the prevalence of reporting items that require more attention, such as randomization details, so essential in this type of study; 3) promote a more balanced distribution in the production of RCTs and scientific collaborations across different regions, especially underrepresented ones. This equity can be facilitated by greater financial support from public and private sources, as well as by encouraging the promotion of open science; and 4) continuing to strengthen the production of RCTs, ensuring that these increasingly higher-quality studies form the basis of clinical decision-making.

In conclusion, our global analysis of RCTs in dentistry in recent years identified essential characteristics of these studies, such as the most frequent journals, the most studied subjects, and citation metrics. We highlight Brazil as the country that produces the most RCTs and the USA as a main collaborator. However, there are few studies and few identified collaborations in countries with low and lower-middle incomes. Finally, we emphasize the variability in the reporting and conduct of studies, with the report of CONSORT use and important randomization data being suboptimal. Attention should be focused on strengthening researchers' knowledge of the appropriate methods for conducting good RCTs and the correct way to report these studies by requiring the use of CONSORT.

## Data Availability

Data, analytical code, or other materials will be shared upon request.

## References

[B1] American Dental Association (2024). Policy Statement on Evidence-Based Dentistry.

[B2] Friedman LM, Furberg CD, DeMets DL, Reboussin DM, Granger CB (2015). Fundamentals of Clinical Trials.

[B3] Zabor EC, Kaizer AM, Hobbs BP (2020). Randomized Controlled Trials. Chest.

[B4] Hung BT, Long NP, Hung le P, Luan NT, Anh NH, Nghi TD (2015). Research trends in evidence-based medicine: a joinpoint regression analysis of more than 50 years of publication data. PLoS One.

[B5] Zhao X, Jiang H, Yin J, Liu H, Zhu R, Mei S (2022). Changing trends in clinical research literature on PubMed database from 1991 to 2020. Eur J Med Res.

[B6] Sarkis-Onofre R, Poletto-Neto V, Cenci MS, Moher D, Pereira-Cenci T (2020). CONSORT endorsement improves the quality of reports of randomized clinical trials in dentistry. J Clin Epidemiol.

[B7] Del Fabbro M, Corbella S, Tsesis I, Taschieri S (2015). The trend of quality of publications in endodontic surgery: a 10-year systematic survey of the literature. J Evid Based Dent Pract.

[B8] Wambier LM, Gonçalves A da R, Wambier DS, Reis A, Chibinski ACR (2022). Adherence to the CONSORT statement of randomized clinical trials on ART restorations in children: current status and reporting characteristics. Braz oral res.

[B9] Schulz KF, Altman DG, Moher D, CONSORT Group (2010). CONSORT 2010 statement: updated guidelines for reporting parallel group randomised trials. BMJ.

[B10] ClinicalTrials.gov (2024). US National Library of Medicine.

[B11] Sterne JAC, Savović J, Page MJ, Elbers RG, Blencowe NS, Boutron I (2019). RoB 2: a revised tool for assessing risk of bias in randomised trials. BMJ.

[B12] Gabriel SE, Normand SL (2012). Getting the methods right--the foundation of patient-centered outcomes research. N Engl J Med.

[B13] The CONSORT Website (2024). CONSORT endorsers- journals.

[B14] Begg C, Cho M, Eastwood S, Horton R, Moher D, Olkin I (1996). Improving the quality of reporting of randomized controlled trials. The CONSORT statement.

[B15] Sponchiado-Júnior EC, Vieira WA, Frozoni M, Herkrath FJ, de-Jesus-Soares A (2021). CONSORT Compliance in Randomized Clinical Trials of Regenerative Endodontic Treatments of Necrotic Immature Teeth: A Scoping Review. J Endod.

[B16] Ortiz MIG, Ribeiro MES, Lima DANL, Silva CM, Loretto SC, da MH (2021). Compliance of randomized clinical trials on dental caries prevention methods with the CONSORT statement: a systematic review. J Evid Based Dent Pract.

[B17] Rezende M, Martins ACR, da Silva JA, Reis A, de Geus JL (2022). Compliance of randomized controlled trials in posterior restorations with the CONSORT statement: a systematic review of methodology. Clin Oral Investig.

[B18] Elagami RA, Reis TM, Hassan MA, Tedesco TK, Braga MM, Mendes FM (2024). CONSORT statement adherence and risk of bias in randomized controlled trials on deep caries management: a meta-research. BMC Oral Health.

[B19] Prado MC, Dotto L, Agostini BA, Sarkis-Onofre R (2023). Metaresearch study highlights the gender gap in randomized controlled trials in dentistry. J Clin Epidemiol.

[B20] Hsiehchen D, Hsieh A, Espinoza M (2019). Prevalence of female authors in case reports published in the medical literature. JAMA Netw Open.

[B21] Council of Dentistry of Brazil (2024). Dental specialties recognized by the Federal Council of Dentistry of Brazil.

[B22] Hutchins BI, Yuan X, Anderson JM, Santangelo GM (2016). Relative Citation Ratio (RCR): A New Metric That Uses Citation Rates to Measure Influence at the Article Level. PLoS Biol.

[B23] Kelaher M, Ng L, Knight K, Rahadi A (2016). Equity in global health research in the new millennium: trends in first-authorship for randomized controlled trials among low- and middle-income country researchers 1990-2013. Int J Epidemiol.

[B24] Lerman TT, Fishman B, Reitblat O, Reitblat T, Goldberg E, Krause I (2021). Global Academic Productivity in the Field of Internal Medicine and Its Correlation to National Economic Indicators: A Bibliometric Analysis of 24 Years. Am J Med Sci.

[B25] Bassani R, Pereira GKR, Page MJ, Tricco AC, Moher D, Sarkis-Onofre R (2019). Systematic reviews in dentistry: Current status, epidemiological and reporting characteristics. J Dent.

[B26] (2022). Scimago Journal and Country Rank.

[B27] Morita MC, Uriarte M, Fontanella VRC, Haddad AE (2020). The unplanned and unequal expansion of Dentistry courses in Brazil from 1856 to 2020. Braz Oral Res.

[B28] Dechartres A, Boutron I, Trinquart L, Charles P, Ravaud P (2011). Single-center trials show larger treatment effects than multicenter trials: evidence from a meta-epidemiologic study. Ann Intern Med.

[B29] Catalá-López F, Aleixandre-Benavent R, Caulley L, Hutton B, Tabarés-Seisdedos R, Moher D (2020). Global mapping of randomised trials related articles published in high-impact-factor medical journals: a cross-sectional analysis. Trials.

[B30] Saltaji H, Armijo-Olivo S, Cummings GG, Amin M, Flores-Mir C. (2017). Randomized clinical trials in dentistry: Risks of bias, risks of random errors, reporting quality, and methodologic quality over the years 1955-2013. PLoS One.

[B31] 3rd Moses H, Matheson DH, Cairns-Smith S, George BP, Palisch C, Dorsey ER (2015). The anatomy of medical research: US and international comparisons. JAMA.

[B32] Catillon M (2019). Trends and predictors of biomedical research quality, 1990-2015: a meta-research study. BMJ Open.

[B33] Koletsi D, Pandis N, Polychronopoulou A, Eliades T (2012). Mislabeling controlled clinical trials (CCTs) as "randomized clinical trials (RCTs)" in dental specialty journals. J Evid Based Dent Pract.

[B34] Furuya-Kanamori L, Xu C, Hasan SS, Doi SA (2021). Quality versus Risk-of-Bias assessment in clinical research. J Clin Epidemiol.

[B35] Berger VW, Bour LJ, Carter K, Chipman JJ, Everett CC, Heussen N (2021). A roadmap to using randomization in clinical trials. BMC Med Res Methodol.

